# Adaptive Immune Response against Hepatitis C Virus

**DOI:** 10.3390/ijms21165644

**Published:** 2020-08-06

**Authors:** Janine Kemming, Robert Thimme, Christoph Neumann-Haefelin

**Affiliations:** 1 Department of Medicine II, Freiburg University Medical Center, Faculty of Medicine, University of Freiburg, Hugstetter Strasse 55, 79102 Freiburg im Breisgau, Germany; janine.kemming@uniklinik-freiburg.de (J.K.); robert.thimme@uniklinik-freiburg.de (R.T.); 2 Faculty of Biology, University of Freiburg, Schaenzlestrasse 1, 79104 Freiburg im Breisgau, Germany

**Keywords:** hepatitis C virus, T cell, B cell, neutralizing antibody, viral escape, T cell exhaustion

## Abstract

A functional adaptive immune response is the major determinant for clearance of hepatitis C virus (HCV) infection. However, in the majority of patients, this response fails and persistent infection evolves. Here, we dissect the HCV-specific key players of adaptive immunity, namely B cells and T cells, and describe factors that affect infection outcome. Once chronic infection is established, continuous exposure to HCV antigens affects functionality, phenotype, transcriptional program, metabolism, and the epigenetics of the adaptive immune cells. In addition, viral escape mutations contribute to the failure of adaptive antiviral immunity. Direct-acting antivirals (DAA) can mediate HCV clearance in almost all patients with chronic HCV infection, however, defects in adaptive immune cell populations remain, only limited functional memory is obtained and reinfection of cured individuals is possible. Thus, to avoid potential reinfection and achieve global elimination of HCV infections, a prophylactic vaccine is needed. Recent vaccine trials could induce HCV-specific immunity but failed to protect from persistent infection. Thus, lessons from natural protection from persistent infection, DAA-mediated cure, and non-protective vaccination trials might lead the way to successful vaccination strategies in the future.

## 1. Introduction

**Figure 1:**
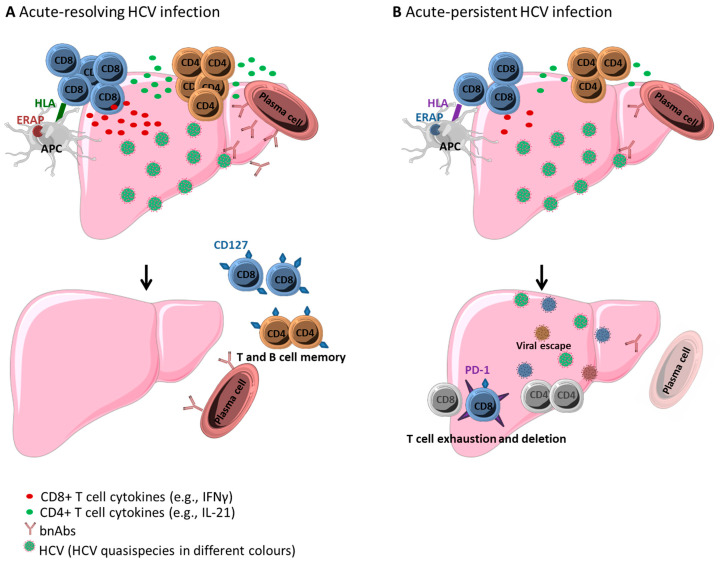
Hepatitis C virus (HCV)-specific adaptive immune response in (**A**) acute-resolving and (**B**) acute-persistent HCV infection. (**A**) In acute-resolving HCV infection, multi-specific, and vigorous HCV-specific CD4+ and CD8+ T cells are primed, and plasma cells produce broadly neutralizing antibodies (bnAbs). After viral clearance, memory cells (expressing, e.g., CD127) are maintained. (**B**) In acute-persistent HCV infection, the initial HCV-specific adaptive immune response is similar to acute-resolving infection, however, CD4+ T cells are rapidly lost, CD8+ T cells exhaust (expressing, e.g., PD-1), and viral escape mutations abrogate recognition by HCV-specific CD8+ T cells and nAbs. Host genetic background, including HLA class I and II alleles, as well as ERAP allotypes, might impact dichotomous outcome. Graphic elements were taken and modified from a Servier Medical Art template licensed under a Creative Commons Attribution 3.0 Unported License (CC BY 3.0) (https://smart.servier.com).

Hepatitis C virus (HCV) has infected approximately 70 million people worldwide. Only a minority of individuals (20–30%) are able to clear the virus spontaneously in the acute phase of infection, while the majority of patients develop persistent infection. These patients are at substantial risk to develop liver inflammation, fibrosis, cirrhosis, and hepatocellular carcinoma (HCC) [1]. Direct-acting antiviral (DAA) treatment regimens revolutionized treatment of chronic HCV infection and now allow cure of nearly all patients treated [1]. Worldwide eradication of HCV infection, however, will most likely require a prophylactic vaccine against HCV, since antiviral treatment of chronically infected patients alone might not hold pace with the rate of new infections, and since re-infection of cured individuals is possible, especially in cohorts with high risk for infection [2]. Recent HCV vaccination trials have failed [3,4,5], and it is thus of utmost importance to define and understand the prerequisites and mechanisms of successful HCV-specific immune responses. In addition, of a more basic immunological perspective, HCV infection is an exciting immunological model, since HCV infection is the only human infection with a dichotomous outcome (viral clearance versus persistence) in a substantial proportion of patients, and the only human chronic infection that can be cured by a well-tolerated drug therapy. It is, thus, a perfect setting to better understand the immunological mechanisms of spontaneous viral clearance, as well as the effects of the loss of antigen on virus-specific immunity in a chronic human viral infection. In the following, we will first address the roles of HCV-specific B cells/neutralizing antibodies, as well as CD4+ and CD8+ T cells, since all of these were demonstrated to have important roles in infection outcome (Figure 1). We will then summarize lessons from successful natural clearance of acute HCV infection, DAA-mediated clearance of chronic HCV infection, and also failed vaccination trials.

## 2. Antibody Response

Early in vitro neutralization studies using immunoglobulin from chronically infected patients as well as active immunization studies using recombinant E1E2 protein generated clear evidence that HCV-specific antibodies can protect chimpanzees from challenge with homologous HCV strains [6,7]. Despite these early findings, the importance of antibodies in HCV infection was underestimated for a long time. This was due to a manifold of reasons [8]. First, reports of successful viral clearance in agammaglobulinemic patients raised doubts regarding the importance of antibodies in protection from persistent HCV infection [9]. Second, HCV infection is associated with the occurrence of several specific and unspecific antibody classes, including antibodies detected by clinical routine serological tests, autoantibodies such as rheumatoid factor involved in extrahepatic manifestations of HCV, and neutralizing antibodies (nAbs). Antibodies detected in clinical routine serology mostly targeting the core and the non-structural proteins, can be detected in (immunocompetent) patients approximately 5–8 weeks post infection (coinciding with the peak of liver enzymes and HCV-specific T cells), and do not correlate with the outcome of infection. Autoantibodies are present in a majority of HCV-infected patients, are a result of B cell dysregulation, and might contribute to extrahepatic manifestations of HCV infection such as mixed cryoglobulinemia. The autoantibody most frequently detected in patients with HCV infection is the anti-immunoglobulin autoantibody rheumatoid factor (RF) that is detectable in approximately 50% of patients with chronic HCV infection and contributes to the production of cryoglobulin [10,11]. Only a small fraction of HCV-specific antibodies have the capability to neutralize viral particles in vitro. The vast majority of these nAbs targets the hypervariable region 1 (HVR1) of the HCV glycoprotein E1. These nAbs are mostly strain-specific, and due to the high mutations rate in the HVR1, resistance against these antibodies develops rapidly, abolishing a protective role of these HVR1-specific nAbs [12,13,14]. Viral diversity is also the third reason for the previous underestimation of the role of antibodies in HCV infection. Indeed, earlier studies on the impact of nAbs in the natural course of HCV infection used viral reference strains as readout in the neutralization assays and did not find a clear correlation between nAbs and viral clearance [15,16].

However, by using autologous viral sequences for such studies, a clear association between early nAb responses and viral clearance could be shown [17,18,19]. These studies also indicated that a distinct subset of nAbs, specifically broadly neutralizing antibodies (bnAbs), correlate with viral clearance. bnAbs have a broad capability to cross-recognize viral quasispecies and strains even from different HCV genotypes. While immunoglobulins from patients with chronic HCV infection can protect animals (humanized mice and chimpanzees, respectively) against homologous but not heterologous HCV challenge [20,21,22], bnAbs could protect animals against both, homologous and heterologous HCV challenge in a large number of studies with a variety of antibodies [23,24,25,26]. A mixture of different bnAbs was even able to abrogate established HCV infection in human liver chimeric mice [27]. bnAbs are, therefore, a current research focus in HCV vaccine development. bnAbs target conformational, discontinuous epitopes on E2 (antigenic regions AR1, AR2, and AR3 including the CD81 binding site involved in HCV cell entry) and the E1E2 heterodimer interface (AR4 and AR5), as well as linear, continuous epitopes on E2 (antigenic sites AS; e.g., AS412 corresponding to E2 amino acid residues 412-423) [8]. The exact binding epitopes of many bnAbs were identified by global alanine scanning of the E1E2 protein, followed by antibody binding assays [28,29]. The immunodominance of bnAbs were analyzed very recently in natural acute infection [30], as well as in samples from a (historic) E1E2 vaccination study in healthy volunteers [31].

Despite these recent advances in the understanding of HCV-specific antibody responses it needs to be kept in mind that antibodies are only the effector molecules of B cells, and that little is known regarding HCV-specific B cells to date. Indeed, the reasons why HCV-specific B cells fail to produce substantial quantities of bnAbs in most patients remain obscure. For example, it was proposed that B cell intrinsic mechanisms as well as a reduced or dysregulated help from CD4+ T cells, especially T follicular helper (Tfh) cells, might contribute to this scenario [32,33]. Recently, the detection of hepatitis B virus (HBV)-specific B cells through flow cytometry was made possible by the use of fluorochrome-coupled HBsAg and HBV core molecules as ‘traits’ [34,35,36,37]. A similar methodological development for HCV-specific B cells, however, is hindered by the lack of knowledge regarding E1E2 structural biology.

In sum, bnAbs are likely an important component in natural HCV clearance and might represent an attractive target for vaccine development. However, new methodological approaches might be necessary to fully understand protective action as well as mechanisms of failure of bnAbs.

## 3. HCV-Specific T Cell Response

For HCV, like for many other viral infections, a functional multi-specific T cell response is essential for viral clearance and prevention of chronicity. Several lines of evidence support the mandatory role of both, CD4+ and CD8+ T cell responses in viral clearance. First, HCV-specific CD4+ and CD8+ T cell responses are temporally tightly linked to the onset of liver disease (increase of liver enzymes, clinical symptoms including jaundice in icteric cases) as well as a sharp decline in viremia [38,39,40]. Second, antibody-mediated depletion of CD4+ as well as CD8+ T cells interfered with viral clearance in the chimpanzee model, the only animal next to humans, which despite not being a natural host could be infected with HCV [41,42]. Of note, after the depletion of CD8+ T cells, viremia was prolonged compared to control chimpanzees, and viremia only declined and the infection finally resolved when the CD8+ T cells reappeared and the HCV-specific CD8+ T cells were detectable [41]. In contrast, CD4+ depletion resulted in persistent viremia that was mechanistically linked to the evolution of viral escape mutations in HCV-specific CD8+ T cell epitopes [42]. These results support the concept that CD8+ T cells are the major antiviral effector cells, while CD4+ T cells provide important help and are thus evenly mandatory for viral clearance. Third, there is also strong immunogenetic evidence for the role of both, CD4+ and CD8+ T cells in HCV clearance, since specific HLA class I and II alleles, restricting CD8+ and CD4+ T cells, respectively, were linked to viral clearance or persistence [43,44,45,46]. Despite these shared and joined key roles in the course of HCV infection, HCV-specific CD4+ and CD8+ differ very much in their nature and magnitude. HCV-specific CD8+ T cell responses are multi-functional and long lasting in acute infection and are maintained even during persistent infection, although they eventually might lose their functionality and change their phenotype. HCV-specific CD4+ T cell responses are initially primed and detectable in all infected individuals, however, they rapidly decline in patients with persistent infection and are hardly detectable once chronicity is established [47,48].

### 3.1. CD8+ T Cell Response in Acute HCV Infection

Following HCV infection, a specific and multifunctional CD8+ T cell response is induced in almost all patients. Primed HCV-specific CD8+ T cells appear in the blood and infiltrate the liver first, after 6–8 weeks post infection [38,39,40]. The reasons for this delay are not known, however, kinetics are similar for T cells targeting other hepatotropic viruses such as hepatitis B virus (HBV). During these initial weeks of HCV infection, viremia is controlled at relatively high levels by the innate immune response, including, e.g., NK cells and type I (IFNα) and III (IFNλ) interferons. The important impact of the innate immune response in viral control is underlined by the association of specific polymorphisms, e.g., the IFNλ system and spontaneous resolution of acute HCV infection [49]. The appearance of the CD8+ T cell response, however, coincides with the onset of liver disease and a drop in viral titers [38,39,40]. HCV-specific CD8+ T cells display an activated phenotype (CD38+) and also display high expression of PD-1, rather indicating activation than exhaustion in this infection phase [39,50]. Of note, HCV-specific CD8+ T cell do not produce antiviral cytokines such as interferon-gamma in the early phase of acute infection, irrespective of infection outcome, a phenotype referred to as “stunned” [40].

Early expression of IL-7 receptor alpha (CD127) and T-bet [51] on HCV-specific CD8+ T cells is linked to successful immune responses, resulting in viral clearance. It is important to note, however, that it has so far not been understood why after an initial priming of HCV-specific CD8+ T cell responses of similar strength and with similar functional and phenotypic characteristics, one individual will clear infection while another progresses to chronic infection. A recent analysis of the early transcriptional differences between HCV-specific CD8+ T cells from patients with acute-resolving versus acute-persistent HCV infection performed by the group of Georg Lauer found a dysregulation of metabolic processes, linked to changes in the expression of genes related to nucleosomal regulation of transcription, T cell differentiation, and the inflammatory response [52]. While the field is far from understanding the complex transcriptional regulation networks that determine the fate of virus-specific T cells, it is intriguing that one of the genes strongly upregulated in resolvers was *TCF7* encoding for the TCF1 protein. High expression of TCF1 is also found on HCV-specific CD8+ T cells that are maintained after successful antiviral treatment of chronic HCV infection (see below, ‘Lessons from DAA therapy’). A gene that was upregulated in patients with viral persistence, however, was p53 [52]. Along with its role in metabolism and carcinogenesis, p53 also has an immune-regulatory role that has recently gained increasing attention. These results were confirmed and extended by the group of Carlo Ferrari, demonstrating that targeting of p53 can rescue impaired glycolytic and mitochondrial functions during early persistent infection [53].

CD8+ T cells also rely on help from CD4+ T cells to perform their full effector function. Thus, absence of CD4+ T cell help might be an important mechanism contributing to viral persistence. Indeed, a weak or impaired HCV-specific CD4+ T cell response with decreased production of IL-2 and IL-21 correlates with a diminished early-phase HCV-specific CD8+ T cell response and viral persistence.

Once HCV is cleared by an effective immune response, CD8+ T cell populations are no longer triggered by ongoing antigen stimulation and start to express high levels of the memory marker CD127, which is needed for homeostatic proliferation, and decline in frequency. However, a robust memory CD8+ T cell response is kept and will rapidly re-expand during reinfection, and might accelerate viral clearance [54]. Despite this memory formation, viral persistence is possible upon reinfection and is almost always associated with the appearance of escape mutations.

### 3.2. CD4+ T cell Response in Acute HCV Infection

During acute infection, HCV-specific CD4+ T cells are primed and initially expand to form a multispecific and multifunctional CD4+ T cell response, irrespective of the outcome of infection. In acute-resolving infection, these CD4+ T cell responses are maintained. In acute-persistent infection, however, these CD4+ cells are rapidly deleted [47,48]. Similar to HCV-specific CD8+ T cells, HCV-specific CD4+ T cells proceed from an activated phenotype with expression of PD-1, CTLA4, and CD38, during acute infection to a memory state, defined by upregulation of CD127 and downregulation of activation markers [55,56], after viral clearance.

### 3.3. Failure of HCV-Specific T Cell Responses in Chronic HCV Infection

The majority of patients are not able to clear acute HCV infection and proceed to chronic HCV infection. The main mechanisms of HCV-specific T cell failure contributing to viral persistence are viral escape and T cell exhaustion. Lack of CD4+ T cell help and production of immunomodulatory cytokines by regulatory T cells (Tregs) [57,58,59,60,61] might further contribute to HCV-specific T cell failure. In addition, impaired function of dendritic cells (DCs) in persistent infection was described very early [62,63,64], however, the precise impact of DC dysfunction on HCV-specific T cell failure remains elusive to date [65].

Viral escape from HCV-specific CD8+ T cell responses typically occurs during the early phase of infection [66,67], with mutations detectable in about 50% of epitopes [67,68], which are associated with viral persistence [67,69,70,71]. Mutations might develop at the HLA class I binding anchors of the epitope, thus, abolishing or lowering the binding affinity of the epitope for the restricting HLA class I molecule, at positions responsible for T cell receptor recognition [72] or at the flanking sites of the epitope, influencing proteasomal processing [70,73,74]. In cases when the evolution of escape mutations is associated with viral fitness cost [72,75,76], the virus might revert to wild-type upon transmission to an individual negative for the restricting HLA class I allele [70]. In addition, compensatory mutations might be required to allow the development of mutations in regions that would otherwise impair viral replication [77,78]. On a populational level, viral escape might lead to HLA class I associated viral sequence polymorphisms (also called HLA class I footprints), since patients positive for the restricting HLA class I allele frequently display the respective mutation in their autologous viral sequences, while patients negative for the restricting HLA class I allele do not [79,80,81,82,83,84,85]. In cases with low viral fitness cost, escape variants might even replace prototype sequences and become the new consensus sequence in a population, resulting in the loss of an HCV-specific CD8+ T cell epitope in the population [86]. Loss of recognition by viral escape might or might not be complete, but priming of de novo T cell responses against mutated epitopes does not occur in persistent HCV infection, possibly due to the lack of CD4+ T cell help and high antigen load during the later stages of persistent infection. Some reports show that HCV-specific CD8+ T cells targeting escaped epitopes still exert some viral control and effector function. This hypothesis is supported by observations showing that if the T cell pressure is attenuated for example during pregnancy, the virus mutates back to its original wild-type sequence. After pregnancy, the CD8+ T cell response is reinvigorated and there is evidence for renewed CD8+ T cell pressure on HLA class I restricted epitopes [87]. However, since the CD8+ T cells targeting escaped epitopes are not exposed to constant T cell receptor triggering anymore, they acquire a memory-like state rather than an exhaustion phenotype with expression of CD127 and sustained proliferative potential [88,89].

In sharp contrast to viral escape, T cells that are exposed to constant cognate antigen stimulation are subject to dramatic changes concerning their phenotype, function, epigenetic, and transcriptional profile [52,90,91], a process termed T cell exhaustion. T cell exhaustion leads to a gradual loss of effector functions ranging from loss of proliferative capacity and cytokine secretion, to loss of cytotoxicity that is accompanied by an upregulation of inhibitory receptors [92,93,94]. Recent studies showed that the exhausted T cells consist of heterogeneous populations, namely, a less exhausted memory like population defined by expression of CD127 and PD-1, and co-expression of TCF1, which is shown to retain the proliferative capacity of these cells and a severely exhausted CD127-PD-1^high^ population [95,96]. This terminally exhausted population shows transcriptional and epigenetic changes that cannot be reversed by antigen removal or anti-PD-1 therapy, whereas the less exhausted cell population can respond to anti-PD-1 therapy [95]. Anti-PD-1 therapy has been examined in the context of chronic HCV infection but showed limited efficacy in chimpanzee and human studies. Indeed, when assessed in a cohort of 54 human patients chronically infected with HCV anti-PD-1, therapy resulted in transiently reduced viremia in a subset of patients, with two patients becoming HCV RNA negative [97]. Out of 3 chimpanzees experimentally infected with HCV, a transient drop in viremia after anti-PD-1 therapy was observed in one animal [98]. Barili et al. could show that exhaustion of HCV-specific CD8+ T cells during chronicity is dominated by a broad gene downregulation, coupled with metabolic and anti-viral function deterioration. The authors succeeded in rescuing the effector functions in these cells by applying histone methyltransferase inhibitors [53].

Terminal T cell exhaustion is also characterized by high expression of the transcription factor Eomes that is in tight balance with its homologue T-bet expressed on progenitor cells of terminally exhausted T cells [99]. In the setting of HCV infection, acute-persistent and chronic infections are characterized by a low frequency of T-bet+Eomes- HCV-specific CD8+ T cells, compared to acute-resolving infection [51]. Recent advances in the field identified the HMG-box transcription factor TOX as crucial for the formation of exhausted T cells. Different groups showed that TOX translates persistent antigen stimulation into a distinct transcriptional and epigenetic program and that in absence of TOX, exhausted T cells do not form [100,101,102,103,104,105]. Noteworthy, deletion of the DNA binding domain of TOX reduced PD-1 expression and increased effector function of T cells, but ultimately these T cells were deleted, indicating that T cell dysfunction and exhaustion is a natural program needed to maintain cell populations that are subject to constant antigen triggering [101]. One of these studies also studied TOX expression on HCV-specific CD8+ T cells. Of note, TOX expression was high on HCV-specific CD8+ T cells, in patients with chronic HCV infection (even after DAA-mediated cure of chronic infection), and these T cells co-expressed CD127, PD-1, and TCF1. HCV-specific CD8+ T cells from patients who spontaneously resolved acute HCV infection, however, displayed low TOX expression, comparable to naïve and influenza-specific CD8+ T cells [101].

Data on viral escape and T cell exhaustion regarding CD4+ T cells in chronic HCV infection is limited, since these cells are readily deleted in persistent infection [47,48]. There is some evidence for viral escape within CD4+ T cell epitopes, but this seems to be rather uncommon overall [106,107,108]. Recent studies using enrichment strategies with antigen specific HLA class II tetramers to overcome low cell numbers, showed that HCV-specific CD4+ T cells indeed express multiple inhibitory receptors like PD-1, TIGIT, and CTLA-4, during chronic infection [47,109]. In in vitro culture, CD4+ T cell functionality could be restored by anti-PD-1 antibody administration, but whether inhibitory receptor expression alone accounts for the deletion of HCV-specific CD4+ T cells is currently unclear. Interestingly, Coss et al. could show an increase of CD4+ T cell functionality and number in a cohort of women after childbirth. This increase correlated with viral control, compared to women in their last trimester and women experiencing no viral control [110]. Previously the same group reported that the reduced viremia was associated with revived CD8+ T cell selection pressure on targeted epitopes [87]. Therefore, the drop in viremia can likely be ascribed to an improved CD4+ T cell functionality, providing CD4+ T cell help and thereby increasing CD8+ T cell effector function.

Treg frequency is enhanced in chronic HCV infection [57,58,60,61]. Important issues regarding Tregs in HCV infection such as antigen-specificity and impact on infection outcome, however, remain elusive to date [59]. Tregs were shown to expand and produce regulatory cytokines such as IL-10 and TGF-β, thereby, potentially interfering with CD4+ and CD8+ T cell immunity, by counteracting inflammatory and activation signals [59]. However, Treg cell number and function in acute infection could not be related to infection outcome [111].

## 4. Lessons from Successful Natural Clearance of Acute HCV Infection

Several HLA class I and II types are associated with spontaneous clearance of acute HCV infection [43,44,45,46]. The mechanisms that contribute to the protective role of the HLA class I types A*03, B*15, B*27, and B*57 were analyzed in detail by the groups of Klenerman et al. (HLA-A*03) [112], Timm et al. (HLA-B*15) [113], Kim et al. (HLA-B*57) [44,78], and our group (HLA-B*27) [76,77,114,115,116,117,118,119]. For all four HLA types, immunodominant HCV-specific CD8+ T cell epitopes were located in E2, the NS3 protease or NS5B polymerase that are targeted in the vast majority of patients with acute HCV infection expressing the respective HLA type were identified [44,112,113,118]. In patients who develop persistent infection, despite expressing the respective protective HLA types, a complex pattern of viral evolution occurs in the immunodominant HCV-specific CD8+ T cell epitopes [76,112]. Indeed, autologous viral sequences from these patients display multiple amino acid mutations within the epitopes, interfering with the recognition of these epitopes by the virus-specific CD8+ T cell responses. Compared to viral epitopes restricted by non-protective HLA types, a single amino acid mutation within the protective epitopes is not sufficient for viral escape from the virus-specific CD8+ T cell response. Rather, several mutations need to occur to (nearly) completely abolish cross-recognition by the epitope-specific T cell responses. Mutations at some positions in the viral epitope, such as the main HLA binding anchors at the position two of the epitope (in the case of HLA-B*27 restricted epitopes an arginine), cannot occur, since the resulting viral variants are not able to replicate at comparable levels, a phenomenon that was termed as the ‘viral fitness cost’. In some cases, mutations within a protective HCV-specific CD8+ T cell epitope even have to be compensated for by an amino acid mutation outside of the epitope, up to 30 amino acids up- or downstream of the epitope, in order to maintain replication levels [77,78,113]. In the setting of acute HCV infection, the HCV-specific CD8+ T cell response targeting these protective epitopes might clear the virus, before this complex pathway of viral escape comprising mutations at several amino acid residues can occur, thus, explaining the high rate of viral clearance associated with these HLA class I types. Next to the functional constraints on the targeted viral epitopes, rapid antigen processing, and thus early priming of dominant virus-specific CD8+ T cell responses might be an additional characteristics of protective HLA class I types such as HLA-B*27 [116]. Targeting such protective HCV-specific CD8+ T cell epitopes might thus be an important aim of HCV-specific prophylactic vaccines. It is important to note that protection by these HLA class I alleles is highly restricted to specific HCV genotypes, subtypes, or even specific strains, as well as specific HLA class I subtypes (alleles). Indeed, HLA-B*27 seems to protect against HCV genotype 1 (1a and 1b), but not genotype 3, a finding that can be explained by the conservation of the immunodominant HLA-B*27 restricted HCV-specific epitope across genotype 1a and 1b, but not other genotypes, including genotype 3a [115]. Similarly, the HLA-B*57 restricted epitope is present in genotype 1a, but not genotype 1b [44]. Even more strikingly, specific infecting strains of the same HCV subtype (1b) display sequence differences in some of these protective HCV-specific CD8+ T cell epitopes, explaining that protective effects of the respective HLA class I types could be demonstrated in one single-source outbreak cohort but not the other [117]. To add complexity even at another dimension, immunodominance of HCV-specific CD8+ T cell responses restricted by HLA-B*27 can also be influenced by precise host genetics, since the HLA-B*27 subtypes (alleles) B*27:05 (representing the ancestral subtype that is also most prominent at the global level) and B*27:02 (a subtype frequently found in the Mediterranean region) do not completely overlap in epitope restriction [119]. In addition, components of the antigen processing/presentation machinery that have so far obtained little attention, such as the endoplasmic reticulum aminopeptidase 1 (ERAP-1) might have a previously underestimated impact on immunodominance, as well as protection in viral infections. ERAP-1 is involved in fine-trimming of antigens to 8-10-mer epitopes that are then ready for presentation by the HLA class I molecules. So far, ERAP-1 was mostly known for the link between ERAP-1 allotypes and HLA-associated autoinflammatory diseases, such as the HLA-B*27-associated ankylosing spondylitis. Of note, however, we could demonstrate that ERAP-1 alloytpes with hyporeactive trimming activity might lead to production and targeting of longer (10- and 11-mer) HCV-specific HLA-B*27-restricted CD8+ T cell epitopes, thus, skewing the usual immunodominance pattern of HLA-B*27-restricted HCV-specific CD8+ T cell epitopes, and thus, likely contributing to the failure of this otherwise protective CD+ T cell responses [114]. In sum, targeting of HCV-specific CD8+ T cell epitopes that have similar characteristics as the immunodominant epitopes restricted by the HLA class I types that protect from viral persistence in the natural course of infection might be an important goal for prophylactic HCV vaccines. However, these epitopes need to be either cross-reactive between different HCV genotypes or the genotype-specific epitopes for each prevalent HCV genotype need to be included in a vaccine.

## 5. Lessons from DAA Therapy

The development of direct acting antiviral (DAA) therapy revolutionized treatment of chronic HCV infection. Current treatment regiments have treatment durations of 8–12 weeks, reach cure rates of 95–100% and are associated with few adverse events. It remains an important issue to monitor the long-term effectiveness of DAA therapy, since very low levels of (intrahepatic) viral replication might lead to recurrence of HCV infection even after several months, especially in the case of rare HCV subtypes or DAA-resistant strains [120]. Next to the great clinical advancement, however, the introduction of DAA therapy has further increased the role of HCV infection as a unique human infection model, since it is the only chronic infection that can be cured by a well-tolerated standard therapy. Thus, it allows to study the impact of antigen removal in patients that were chronically infected for decades [121]. A first study by our laboratory showed an increase of the ex vivo frequency of HCV-specific CD8+ T cells as well as a restored proliferative capacity of these CD8+ T cells [122]. Further analysis demonstrated that this partial functional restoration of the HCV-specific CD8+ T cell response during and after DAA-mediated viral clearance was due to the maintenance of a memory-like T cell subset that co-expressed the memory marker CD127, as well as the exhaustion/activation marker PD-1, and was further characterized by expression of the transcription factor TCF1. In contrast, terminally exhausted CD127-PD-1^high^TCF1- HCV-specific CD8+ T cells disappeared after HCV elimination. Upon re-challenge with HCV, memory-like CD127+PD-1+TCF1+ HCV-specific CD8+ T cells expand and give rise to the re-emergence of terminally exhausted CD127-PD-1^high^TCF1- HCV-specific CD8+ T cells [96]. Interestingly, the memory-like phenotype of HCV-specific CD8+ T cells is not only observed in the case of DAA-mediated viral clearance, but also in the case of viral escape, interfering with antigen recognition by the epitope-specific CD8+ T cells [96]. It is important to note that memory-like HCV-specific CD8+ T cells that are maintained and enriched after DAA-mediated cure are different from “conventional” memory HCV-specific CD8+ T cells observed in patients, after spontaneous resolution of acute HCV infection, indicated by a substantially higher co-expression of CD127 and PD-1, a higher expression of Eomes, and a lower expression of TCF1 [96]. In line with this partial phenotypical recovery, HCV-specific CD8+ T cells partially recover in function, such as IFNγ and TNF production, but are not fully restored to the level of conventional memory CD8+ T cells found in patients with spontaneously resolved acute HCV infection [96]. Of note, these phenotypical and functional impairments that remain after DAA-mediated cure of chronic HCV infection are also associated with sustained metabolic impairments such as mitochondrial dysfunction [123] and might be more severe in patients with advanced liver disease as well as male patients [123]. These data collectively indicate that HCV-specific CD8+ T cells develop defects during chronic infection that cannot simply be restored by antigen removal. This concept is supported by the current finding that expression of TOX, a central transcription factor regulating T cell exhaustion, remains high after DAA-mediated cure of chronic infection, while TOX is not expressed by HCV-specific CD8+ T cells after spontaneous clearance of acute HCV infection [101]. These findings mimic the situation in the LCMV mouse model, where chronic infection leads to irreversible TOX expression, probably due to epigenetic programming [100,101]. This concept is also in line with the finding that restoration of HCV-specific CD8+ T cells is possible by antiviral treatment early in infection (e.g., acute HCV infection) [124], and the observation in mice that virus-specific CD8+ T cells can be rescued from differentiation to exhausted T cells by antigen removal, early but not late in LCMV infection [125]. This persistent defect of HCV-specific CD8+ T cells might contribute to the lack of protection against re-infection after DAA-mediated cure of chronic HCV infection. Indeed, in the chimpanzee model, viral persistence developed after re-infection, despite the intrahepatic presence of HCV-specific CD8+ T cells primed during the primary infection. This finding could be explained by the persistence of phenotypical alterations (low CD127 expression, high PD-1 expression) found on intrahepatic HCV-specific CD8+ T cells, even two years after DAA-mediated viral clearance [126]. It is thus a major research priority to further define the epigenetic regulation of sustained defects in HCV-specific CD8+ T cells, after DAA-mediated cure. Novel targets might be needed to overcome HCV-specific CD8+ T cell failure after HCV cure and thus protect individuals at continued risk from re-infection.

HCV-specific CD4+ T cells remain at a very low frequency and with a dysfunctional phenotype after DAA-mediated HCV cure [127]. In addition, frequencies of regulatory T cells remained at increased levels after cure [128]. Of note, however, an HCV-specific CD4+ T cell subset with follicular T helper (Tfh) cell signature was maintained during and in the long-term, after DAA-mediated viral clearance. This Tfh cell subset was also responsible for a temporary increase of the CD4+ T cell frequency at week two of DAA therapy, which was most likely due to the efflux of liver infiltrating Tfh cells into the peripheral blood, following virus elimination [32]. HCV-specific Tfh cells might represent an important target population for preventive vaccination strategies.

## 6. Lessons from Vaccine Trials

There are many challenges to HCV vaccine design [3,4,129]. Although HCV infection can be cleared in about 30% of patients in the acute phase of infection, the exact correlates of viral persistence versus resolution are obscure. As discussed in detail above, many studies demonstrated that viral clearance is associated with an early, vigorous, broadly directed, functional, and sustained CD4+ and CD8+ T cell response, as well as with the early appearance of broadly neutralizing antibodies, but the exact mechanisms that lead to an induction of this favorable immune response remain unknown. Additionally, the genetic heterogeneity of HCV is hard to address. Seven HCV genotypes circulate world-wide and each can be further separated into numerous subtypes. In addition, the error-prone RNA-dependent RNA polymerase activity leads to the generation of innumerable quasispecies within a single host, allowing viral escape from the host immune response and further complicating vaccine development. HCV cannot be kept in cell culture easily, making the generation of live attenuated or killed modified virus vaccines extremely difficult. Next to humans, only chimpanzees can persistently be infected with HCV. Other non-human primates such as tree shrews (*Tupaia belangeri*) can be infected with HCV, but develop only transient viremia, indicating that their use in vaccination studies requires further optimization of the model [130]. Immune-competent small animal models of HCV infection were only recently established [131,132]. HCV-naïve individuals at high risk for infection such as people who inject drugs (PWID) are optimal candidates for HCV vaccine efficacy trials, but these cohorts are rare and need intensive care to be maintained [133,134].

Current vaccine strategies do not aim to prevent HCV infection (sterilizing immunity), but rather have the goal to prevent viral persistence upon infection (protective immunity). Two different vaccination strategies are under evaluation for inducing protective immunity. Vaccines aiming to induce broadly neutralizing antibodies (bnAbs) and vaccines aiming to induce protective CD4+ and CD8+ T cell responses.

Many different vaccination strategies were evaluated in order to induce bnAb responses, however, the large majority of these vaccines did not advance to a clinical stage. Most promising results from pre-clinical studies were obtained for the recombinant full-length E1E2 protein from a single genotype 1a strain, with an oil–water adjuvant. This vaccine led to the formation of bnAbs and reduced rates of viral persistence in rodents, primates, and chimpanzees [6,135]. However, it failed to induce antibodies in the majority of patients in a phase 1a human trial [136,137]. Vaccination strategies based on bnAbs might thus have a long road ahead before they show promising clinical effects. Indeed, based on recent advances in the definition of bnAb epitopes discussed above, more targeted antigens than a full-length E1E2 protein just from a single HCV strain might be more effective; in addition, novel strategies to adjuvant the HCV antigen are likely to enhance the chance to induce substantial bnAb levels. Last but not least, an overwhelming amount of data from the natural course of HCV infection indicates that a humoral immune response alone is unlikely to achieve viral clearance in a substantial proportion of infected individuals, suggesting that vaccines designed to induce bnAbs should be used in combination with vaccines designed to induce a protective CD4+ and CD8+ T cell response.

A variety of strategies were used to induce HCV-specific CD4+ and CD8+ T cells in animal models [3,4,129]. Most of these studies focused on the non-structural HCV proteins (NS3, NS4A, NS4B, NS5A, NS5B), since these proteins are more conserved and more often targeted by HCV-specific T cells, compared to the envelope glycoproteins. While most vaccines were able to induce HCV-specific CD4+ and CD8+ T cell responses of variable functionality at least in a subset of animals, only few vaccines were further tested for their ability to protect chimpanzees from persistent HCV infection. Chimpanzees are the only primates that can be chronically infected with HCV next to humans and served as an HCV infection model, until these experiments were abandoned due to ethical concerns. Most, but not all, of these chimpanzee vaccine studies demonstrated reduced rates of HCV persistence in vaccinated versus control animals [138]. Encouraging results were obtained for a vaccination strategy based on replication-defective adenoviral vectors encoding the non-structural HCV proteins (NS3-NS5B) [139]. In the initial chimpanzee study, human adenovirus (Ad) serotypes 6 and 24 were used as vectors, since neutralizing antibodies against these two adenovirus serotypes have a low seroprevalence in humans, and HCV genotype 1b was used as a viral sequence. After priming with Ad6 and boosting with Ad24, an additional boosting was performed with electroporated plasmid DNA. Upon challenge with HCV genotype 1b, all five vaccinated chimpanzees displayed substantially lower viral titers, compared to the control animals, and four of five chimpanzees cleared the infection after a significantly shorter duration of viremia, compared to the control animals, while one vaccinated chimpanzee developed persistent infection [139]. Further immunological analysis demonstrated an early expansion of CD8+ T cells with high CD127 expression, low PD-1 expression, and increased effector function, compared to the control animals developing persistent infection [140]. Strikingly, early expansion of CD8+ T cells with high expression of CD127 and high functionality was also identified as a hallmark of spontaneous clearance of acute HCV infection in the chimpanzee model [141]. Based on these results, the vaccination strategy was further adapted and tested in healthy volunteers not at risk for HCV infection [142,143]. In order to further minimize problems related to preexisting or primed Ad-specific neutralizing antibodies, an Ad6 prime, chimpanzee adenovirus 3 (ChAd3) boost regimen was used in the first human trial [142], and this was further optimized by the use of a ChAd3 prime, modified vaccinia Ankara (MVA) boost regimen, with improved boosting capacity [143]. This vaccination strategy induced vigorous, multispecific, and polyfunctional CD8+ T cells, mostly central and effector memory T cells that expressed CD127, but not PD-1, and were sustained for at least one year. Based on these promising data, the first and so far only clinical trial in individuals at high risk for HCV infection was performed in the US. The double-blind, randomized, placebo-controlled phase I/II study assessed the efficacy of the ChAd3-HCV1b-NS prime and MVA-HCV-1b-NS boost vaccination regimen in a large PWID cohort of 548 HCV-naïve individuals that was completed in 2019. Unfortunately, the vaccine could not prevent chronic HCV infection when compared to the unvaccinated control cohort, with 14/273 individuals developing chronic HCV infection in the vaccine group versus 14/275 individuals in the placebo group [5]. While these results are overall disappointing, it is important to point out that 78% of vaccinated trial participants generated T cell responses to one or more vaccine antigen pools. In addition, individuals who were vaccinated and infected displayed significantly lower peak viral titers (approximately 5-fold) than those who received placebo. These results allowed the interpretation that the vaccine induced T cell responses that were able to control viremia, at least partially. The long-term failure of these vaccine-induced T cell response might either indicate that the T cell responses were not vigorous enough, calling, for example, for a more effective adjuvant, or that cross-recognition of HCV genotypes, subtypes, or even quasispecies circulating in the US by the vaccine-induced T cell response was not sufficient. Indeed, CD8+ T cell epitopes that were induced by this vaccination regimen and targeted immunodominant HCV-specific epitopes displayed a limited capacity to cross-recognize viral variants circulating in the population [144]. This interpretation is also in line with the finding that the epitope repertoire between HCV genotype 1 and genotype 3 or 4, respectively, display little overlap [145,146]. Thus, current research addresses novel adjuvant formulations such as the use of MHC class II invariant chain-adjuvanted viral vectors, enhancing the peak magnitude, breath, and proliferative capacity of HCV-specific T cells induced by the ChAd3-HCV1b-NS prime/MVA-HCV-1b-NS boost vaccine in healthy volunteers [147]. In addition, the team of Eleanor Barnes further optimized the vaccine strategy to generate pan-genotypic T cell responses to conserved subdominant epitopes [148]. For this purpose, only viral sequence regions with a high grade of conservation between the major HCV genotypes (1 and 3 or 1-6, respectively) were included in the vaccine, and this vaccine was also adjuvanted by MHC class II invariant chain [149]. In a mouse model, this strategy clearly enhanced the magnitude, breath, and cross-reactivity of vaccine-induced T cell responses [149]. So far, however, it is not clear if this in vitro advantage will also translate to protective immunity in individuals at risk. Indeed, immunodominant HCV-specific CD8+ T cell epitopes restricted by protective HLA class I types such as HLA-A*03, B*27, and B*57 show little conservation between HCV genotypes or even subtypes [44,112,115,117]. These ‘protective’ epitopes are thus excluded from the vaccine that is engineered to cover only highly conserved HCV sequence regions.

In conclusion, a future efficacious vaccine will likely have to induce cell-mediated as well as humoral immunity. For achievement of this goal, further research on cross-reactive epitopes, conserved regions within the HCV genome, correlates of protective immunity, and the role of bnAbs is urgently needed.

## 7. Conclusions

Global elimination of HCV infection will most likely depend on a prophylactic HCV vaccine. During the last few years, great progress was made in the understanding of successful HCV-specific immunity in acute-resolving HCV infection, as well as the mechanisms of HCV-specific CD8+ T cell failure in persistent infection. In addition, the great clinical advance of DAA therapy allowing cure of nearly all patients with chronic HCV infection enabled the analysis of partial restoration of HCV-specific immunity after clearance of the chronic infection. These new insights into HCV immunobiology, together with lessons from recently failed HCV vaccine trials might lead the way to successful vaccination strategies for both, individuals at risk for primary infection, as well as re-infection after DAA-mediated cure.

## Abbreviations

Ad	adenovirus
AR	antigenic region
AS	antigenic site
(b)nAb	(broadly) neutralizing antibody
CD	cluster of differentiation
ChAd	chimpanzee adenovirus
CTLA4	cytotoxic T lymphocyte antigen 4
DAA	direct-acting antivirals
E1E2	envelope 1/2 protein
ERAP	endoplasmic reticulum aminopeptidase
HBsAg	Hepatitis B surface antigen
HBV	hepatitis B virus
HCC	hepatocellular carcinoma
HCV	hepatitis C virus
HLA	human leukocyte antigen
HMG	high mobility group
HVR	hypervariable region
IFN	interferon
MVA	modified vaccinia Ankara
NS	non-structural (protein)
PD-1	programmed death 1
PWID	people who inject drugs
TCF	T cell factor
Tfh	T follicular helper (cell)
TIGIT	T-cell immunoreceptor with Ig and ITIM domains
TNF	tumor necrosis factor
TOX	thymocyte selection-associated HMG box
